# The complete plastid genome of *Bactris riparia* (Arecaceae) and a comparative analysis in Bactridinae (Cocoseae, Arecaceae)

**DOI:** 10.1590/1678-4685-GMB-2021-0305

**Published:** 2022-11-07

**Authors:** Micheli Cristina Dias, Charles Roland Clement, Hugo Pacheco de Freitas Fraga, Raquel Santos da Silva, Doriane Picanço Rodrigues, Leila do Nascimento Vieira

**Affiliations:** 1 Universidade Federal do Paraná, Departamento de Botânica, Curitiba, PR, Brazil.; 2 Instituto Nacional de Pesquisas da Amazônia, Coordenação de Tecnologia e Inovação, Manaus, AM, Brazil.; 3 Universidade Federal do Amazonas, Departamento de Biologia, Manaus, AM, Brazil.

**Keywords:** Palms, Amazonian floodplains, plastome, molecular evolution

## Abstract

Here we sequenced and characterized the complete plastome of *Bactris riparia*, a species closely related to *B. gasipaes* and widely distributed in Western Amazonia. We performed a comparative genomic analysis with *B. riparia* and the other four Bactridinae species retrieved from GenBank. The plastome of *B. riparia* was 156,715 bp with a quadripartite structure. Gene content included 86 protein-coding genes (CDS), 38 tRNAs, and 8 rRNAs. *Bactris riparia* has 69 more base pairs than *B. gasipaes*, with identical numbers in IR, and more in LSC and SSC. The comparative analysis indicated that structure, collinearity, and IR/SSC borders of plastomes within subtribe Bactridinae are, in general, conserved. We predicted 69 SSRs in *B. riparia* plastome. Among them, ~80% consisted of A/T homopolymers. Among the 52 variable CDS, *rbcL* showed the highest non-synonymous rate, while the *rps15* gene had the highest synonymous rate. Three genes (*ccsA*, *cemA*, and *rpoC1*) presented evidence of positive selection and 22 genes showed evidence of purifying selection. The phylogenetic tree based on plastome sequences set *Bactris* as more closely related to *Astrocaryum* than to *Acrocomia*. These new plastome data of *B. riparia* will contribute to studies about the diversity, evolutionary history, and conservation of palms.

Palms (family Arecaceae), icons of tropical landscapes, form an economically and ecologically important group of ~2,500 species with remarkable diversity and abundance in the Neotropics (Dransfield et al., 2008). These species provide valuable ecosystem services, such as provision (food, medicines, and raw materials), support and regulation (contributing to the maintenance of biodiversity), and cultural (leisure and spirituality of various human groups) (Cámara-Leret et al., 2014). Nonetheless, their phylogenetic diversity has not been safeguarded under current and possible future climate conditions by the existing network of Protected Areas (Velazco et al., 2020).

Given such relevance, palms have become a model group for studies on tropical forests (Faurby et al., 2016). However, as most of these efforts focused on the relationship of major clades of Arecaceae, the phylogenetic relationships at lower taxonomic levels require refinement (Baker and Dransfield, 2016). For example, *Bactridinae* (Cocoseae, Arecaceae) consists of five genera (*Acrocomia* Mart., *Aiphanes* Willd., *Astrocaryum* G. Mey., *Bactris* Jacq. ex Scop., and *Desmoncus* Mart.), about 150 species, and represents approximately 20 % of the Neotropical palm flora. The subtribe is well-characterized by the presence of epidermal spines and by adherent fibers and deep pores in the endocarp (Dransfield *et al*., 2008), but its intergeneric evolutionary relationships require more study (Baker and Dransfield, 2016).

Previous studies sought to elucidate the phylogenetic relationships within the subtribe using multiple nuclear and plastid markers (Eiserhardt et al., 2011). However, they did not yield well-defined and supported relationships (Baker and Dransfield, 2016). Due to structural and evolutionary particularities, the plastome has become a valuable tool for taxonomic, phylogenetic, biogeographic, population genetics, and biotechnological studies (Rogalski et al., 2015). We sequenced and characterized the plastome of the Amazonian floodplain palm *Bactris riparia* Mart., a close relative of the economically important *Bactris gasipaes* Kunth (Couvreur et al., 2007; Clement et al., 2017), and performed comparative analyzes in the subtribe Bactridinae. For the taxonomic review of the genus *Bactris* see Henderson (2000).

For this, fresh leaf tissue of *Bactris riparia* was collected from a single individual (SisGen register number AB0ED60) close to the Solimões River in Manacapuru (AM, Brazil). A sample was deposited in the INPA herbarium, under accession number 290838. The genomic DNA was extracted using DNeasy Plant Mini Kit (Qiagen, Valencia, CA, USA). The DNA was quantified using Qubit fluorometer (Thermo Fisher Scientific) and DNA quality was verified by spectrometry using Nanodrop 2000 (Thermo Fisher Scientific) and by agarose gel electrophoresis (0.8 %, GelRed-stained^TM^). Libraries were prepared with genomic DNA (100 ng) using Illumina DNA Prep kit (Illumina, San Diego, CA, USA), and sequenced on an Illumina HiSeq 2500 platform (Illumina Inc.) using a V4 chemistry at NGS Soluções Genômicas (Piracicaba, Brazil). The resulting paired-end reads (2 x 100 bp) were *de novo* assembled using NOVOPlasty (Dierckxsens et al., 2016). The annotation of the plastome gene content was performed using Geneious Prime® 2021.1.1 (Biomatters Ltd., San Diego, CA, USA) and manually verified using *Bactris gasipaes* (MW054718) as reference. The plastome sequence was submitted to GenBank (MZ823390). The comparative analyzes involved *Bactris riparia* and four species of Bactridinae available in GenBank: *Acrocomia aculeata* (Jacq.) Lodd. ex Mart. (NC_037084.1), *Astrocaryum aculeatum* G. Mey (NC_044482.1), *Astrocaryum murumuru* Mart. (NC_044481.1), and *Bactris gasipaes* Kunth (MW054718). Possible structural rearrangements were verified using the progressive algorithm of Mauve 2.4.0 (Darling et al., 2004). The four plastome junctions (IRb/LSC, IRb/SSC, SSC/IRa, IRa/LSC) were analyzed and illustrated by IRScope (Amiryousefi et al., 2018). Simple sequence repeats (SSRs) were detected using MISA-web (Beier et al., 2017) with thresholds of ten repeat units for mononucleotide SSRs, six repeat units for di- and trinucleotide SSRs, and five repeat units for tetra-, penta-, and hexanucleotide SSRs. The maximum length of sequence between two SSRs to register as compound SSR was set to 100. The 79 protein-coding genes of Bactridinae species (*Bactris riparia*, *Acrocomia aculeata*, *Astrocaryum aculeatum*, *Astrocaryum murumuru*, and *Bactris gasipaes*) were extracted using Geneious Prime and individually align with the aid of MAUVE implemented on Geneious Prime. Among them, 27 genes were 100% identity and were not used for selective pressure analysis. The numbers of non-synonymous and synonymous substitutions rate were estimated using DNASP v6.12.03. Then, the Ka/Ks ratio was estimated. For the phylogenetic analysis, we used the plastomes of the five species of Bactridinae and three species as outgroup (*Cocos nucifera* L. KF285453, *Elaeis guineensis* Jacq. NC_017602, and *Syagrus coronata* (Mart.) Becc. NC_029241) to obtain a multiple alignment (one IR excluded) on Mauve 2.0. The locally collinear blocks (LCBs) from the Mauve alignment were extracted and concatenated into a single alignment using Geneious Prime®. Subsequently, the resulting alignment was submitted to Model Selection implemented in W-IQ-TREE (Trifinopoulos et al., 2016), and the algorithm suggested the best evolutionary model for our data set was HKY+F+I model, according to the Bayesian Information Criterion (BIC). Then, the phylogenetic dendrogram was generated with W-IQ-TREE (Trifinopoulos et al., 2016), under the Maximum Likelihood (ML) probabilistic model and 1000 bootstrap replications.

A total of 55 million reads were generated by the Illumina Hiseq 2500 platform, of which 378,906 reads were used in the assembly of the *Bactris riparia* plastome with an average depth of 268×. The assembled plastome was 156,715 bp in length and presented the typical quadripartite structure of angiosperms (LSC - 85,153 bp; IRs - 27,038 bp; SSC - 17,486 bp). The GC content was 37.5 %, consistent with what was expected for Bactridinae plastomes (Table 1). The gene content is conserved among Bactridinae, with a total of 132 genes, including 86 protein-coding genes, 38 tRNAs and 8 rRNAs (Table 1). The *ndhD* and *rpl2* genes had the unusual ACG start codon, which was shown to be corrected by the post-transcriptional process of mRNA editing involving the pentatricopeptide repeat (PPR) proteins (Boussardon et al., 2012). The *cemA* gene exhibited the same alternative start codon reported for *B. gasipaes,* distinct to the other species of Bactridinae sequenced so far (*Astrocaryum aculeatum*, *A. murumuru*, and *Acrocomia aculeata*) (Lopes et al., 2019; Santos da Silva et al., 2021).

**Table 1-:** General features of Bactridinae plastid genomes.

Plastome Features	*Acrocomia aculeata*	*Astrocaryum aculeatum*	*Astrocaryum murumuru*	*Bactris gasipaes*	*Bactris riparia*
Plastome length (bp)	155,829	156,804	156,801	156,646	156,715
LSC (bp)	84,265	85,037	85,017	85,118	85,153
IR (bp)	27,092	27,081	27,081	27,038	27,038
SSC (bp)	17,380	17,605	17,622	17,452	17,486
GC (%)	37.5	35.4	37.4	37.8	37.5
Gene number	133	133	133	133	133
CDS	86	86	86	86	86
tRNA	38	38	38	38	38
rRNA	8	8	8	8	8

The comparative analysis of the IRs junctions within species of Bactridinae revealed a highly conserved region. The length of the IRs ranged from 27,038 to 27,092 bp. *Bactris gasipaes* and *Bactris riparia* have IRs with the same length (27,038 bp) (Table 1). All Bactridinae plastomes exhibited the *trnH-rps19* gene cluster and showed the incorporation of part of the *ndhF* gene into IRb. An expansion of IRa over SSC was observed in the *ycf1* gene, which appears as a pseudogene in IRb and with an overlap of 56 bp with *ndhF*, a characteristic also present in other species of the tribe Cocoseae (de Souza Magnabosco et al., 2020). There was a marked intergeneric divergence when comparing the length of the *rpl22*-*rps19* (IRb/LSC) and *rps19*-*psbA* (IRa/LSC) intergenic spacers (Figure 1). These junctions are widely described in the literature as regions of greater dynamism among angiosperm plastomes (Zhu et al., 2016). The IRs are the most conserved elements of plastomes, and the rate of synonymous substitutions in IRs is on average 3.7 times lower than in SSC due to a dose-dependent effect (Zhu *et al*., 2016). However, the borders between the IRs and SSC are more dynamic and during evolution they have gone through several lineage-specific contraction and expansion events (Goulding et al., 1996).

**Figure 1-:**
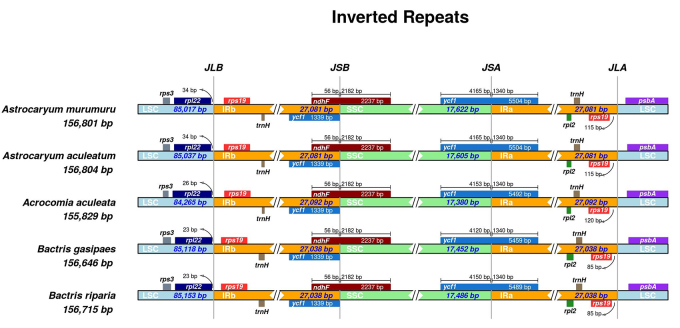
Plot of Bactridinae plastomes comparing the IR junctions sites. Genes transcribed clockwise and counterclockwise are shown above and below of their corresponding tracks, respectively. JLB - IRb/LSC junction; JSB - IRb/SSC junction; JSA - SSC/IRa junction; and JLA - IRa/LSC junction.

The collinearity analysis indicated the conservation of most of the plastome, as shown by the three LCBs identified by Mauve alignment (Figure 2). *Bactris riparia* and *B. gasipaes* are identical in gene content and order, with no rearrangements identified. Divergence was verified in *A. murumuru* and *A. aculeatum*, in which there is an inversion of 4.6 kb. This lineage-specific rearrangement, described by Lopes et al. (2019), is from a flip-flop recombination between 28 bp inverted repeats, which flank the 4.6 kb inversion and is in the *trnT*-UGU/*ndhC* and *trnL*-UAA/*trnV*-UAC intergenic spacers.

**Figure 2-:**
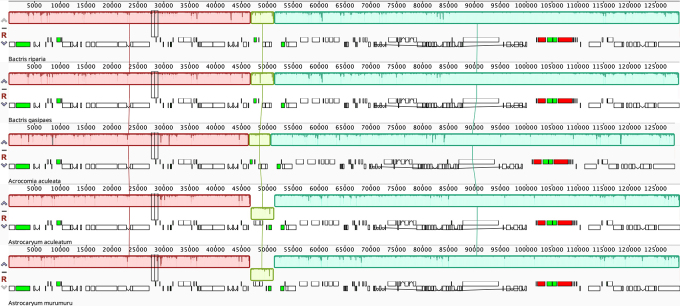
Multiple alignment on Mauve comparing species of Bactridinae. Colors in the alignment represent the Locally Collinear Blocks (LCBs). Annotated genes are shown as boxes below the alignment where annotated CDS features are shown as white boxes, tRNAs are green, rRNAs are red.

The prediction of SSRs revealed a total of 69 SSRs. Among them, homopolymers were the most common (61 occurrences), followed by dipolymers (7 occurrences) and tetrapolymers (1 occurrence). Among the 61 monolymers, 57 were consisted of A/T sequences and all presented 15 repeats or less, which is in accordance to the nature of plastid SSRs of generally <15 mononucleotide repeats. Tri-, penta- and hexapolymers were not present according to the analysis settings used here.

The synonymous (Ks) and non-synonymous (Ka) substitution rates, and the ratio (Ka/Ks) were calculated for 52 genes. Among them, 27 genes had no changes in the synonymous or nonsynonymous rates (Table S1). The *rbcL* gene showed the highest non-synonymous rate (Ka = 0.00546), while the *rps15* gene had the highest synonymous rate (Ks = 0.02266). Three genes (*ccsA*, *cemA*, and *rpoC1*) presented evidence of positive selection (Ka/Ks ratio > 1.0) and 22 genes showed evidence of purifying selection (Ka/Ks ratio < 1.0; Table S1).

The phylogenetic inference of Bactridinae by ML consisted of an alignment of eight sequences with 133,566 columns, among which were 718 distinct patterns, 787 parsimony-informative sites, 972 singleton sites, and 131,807 constant sites. The resulting phylogeny presented highly supported nodes and intergeneric relationships (Figure 3). The topology obtained here corroborated the well-reported monophyly of the subtribe Bactridinae and the genus *Bactris* (Eiserhardt et al., 2011). It also shows *Bactris* as more closely related to *Astrocaryum* than to *Acrocomia*, which was previously recorded by Eiserhardt *et al*. (2011), Faurby et al. (2016) and Santos da Silva et al. (2021). The positioning of *Acrocomia* in Bactridinae has presented a series of inconsistencies over the years, which could be elucidated by a more complete sampling of Bactridinae plastomes, including *Aiphanes* and *Desmoncus*.

**Figure 3-:**
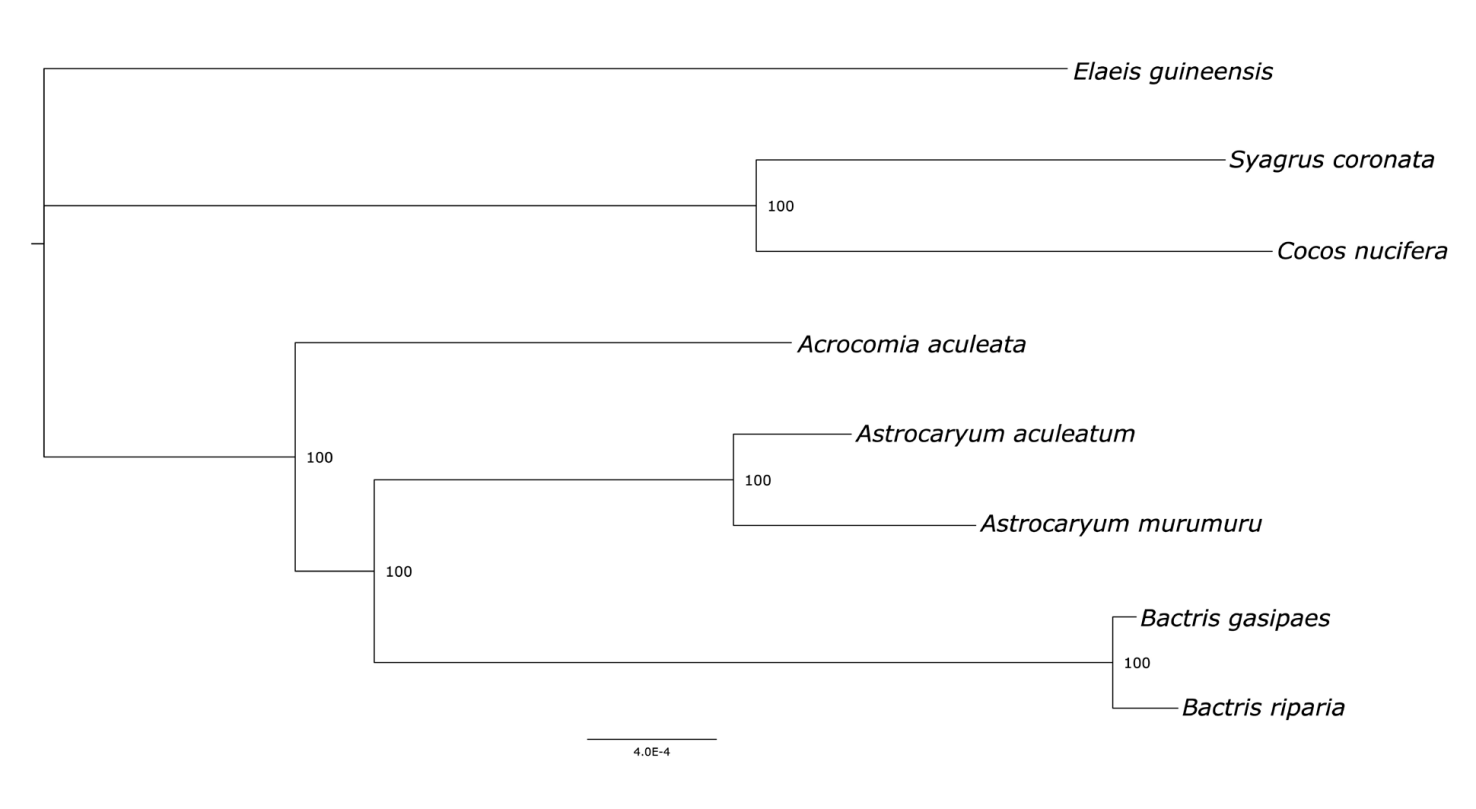
Phylogenetic tree based on Maximum Likelihood using complete plastomes. Numbers above branches are maximum likelihood bootstrap values (1,000 replicates).

## Supplementary material

The following online material is available for this article:

Table S1 - Non-synonymous (Ka), synonymous (Ks), and Ka/Ks ratio of Bactridinae protein-coding genes.

## References

[B1] Amiryousefi A, Hyvönen J, Poczai P (2018). IRscope: An online program to visualize the junction sites of chloroplast genomes. Bioinformatics.

[B2] Baker WJ, Dransfield J (2016). Beyond Genera Palmarum: Progress and prospects in palm systematics. Bot J Linn Soc.

[B3] Beier S, Thiel T, Münch T, Scholz U, Mascher M (2017). MISA-web: A web server for microsatellite prediction. Bioinformatics.

[B4] Boussardon C, Salone V, Avon A, Berthomé R, Hammani K, Okuda K, Shikanai T, Small I, Lurin C (2012). Two interacting proteins are necessary for the editing of the ndhD-1 site in Arabidopsis plastids. Plant Cell.

[B5] Cámara-Leret R, Paniagua-Zambrana N, Balslev H, Barfod A, Copete JC, Macía MJ (2014). Ecological community traits and traditional knowledge shape palm ecosystem services in northwestern South America. For Ecol Manage.

[B6] Clement CR, Cristo-Araújo M, Coppens d’Eeckenbrugge G, Reis VM, Lehnebach R, Picanço-Rodrigues D (2017). Origin and dispersal of domesticated peach palm. Front Ecol Evol.

[B7] Couvreur TL, Hahn WJ, Granville JJD, Pham JL, Ludena B, Pintaud JC (2007). Phylogenetic relationships of the cultivated Neotropical palm Bactris gasipaes (Arecaceae) with its wild relatives inferred from chloroplast and nuclear DNA polymorphisms. Syst Bot.

[B8] Darling ACE, Mau B, Blattner FR, Perna NT (2004). Mauve: Multiple alignment of conserved genomic sequence with rearrangements. Genome Res.

[B9] de Souza Magnabosco JW, de Freitas Fraga HP, da Silva RS, Rogalski M, de Souza EM, Guerra MP, Vieira LDN (2020). Characterization of the complete plastid genome of Butia eriospatha (Arecaceae). Genet Mol Biol.

[B10] Dransfield J, Uhl NW, Lange CBA, Baker WJ, Harley MM, Lewis CE (2008). Genera Palmarum: The Evolution and Classification of Palms.

[B11] Dierckxsens N, Mardulyn P, Smits G (2016). NOVOPlasty: De novo assembly of organelle genomes from whole genome data. Nucleic Acids Res.

[B12] Eiserhardt WL, Pintaud JC, Asmussen-Lange C, Hahn WJ, Bernal R, Balslev H, Borchsenius F (2011). Phylogeny and divergence times of Bactridinae (Arecaceae, Palmae) based on plastid and nuclear DNA sequences. Taxon.

[B13] Faurby S, Eiserhardt WL, Baker WJ, Svenning JC (2016). An all-evidence species-level supertree for the palms (Arecaceae). Mol Phylogenet Evol.

[B14] Goulding SE, Wolfe KH, Olmstead RG, Morden CW (1996). Ebb and flow of the chloroplast inverted repeat. Mol Gen Genet.

[B15] Henderson A (2000). Bactris (Palmae). Organization for Flora Neotropica.

[B16] Lopes AS, Pacheco TG, Silva ON, Cruz LM, Balsanelli E, Souza EM, Pedrosa FO, Rogalski M (2019). The plastomes of Astrocaryum aculeatum G. Mey. and A. murumuru Mart. show a flip-flop recombination between two short inverted repeats. Planta.

[B17] Trifinopoulos J, Nguyen L, Haeseler A, Minh BQ (2016). W-IQ-TREE: A fast online phylogenetic tool for maximum likelihood analysis. Nucleic Acids Res.

[B18] Rogalski M, do Nascimento, Vieira L, Fraga HP, Guerra MP (2015). Plastid genomics in horticultural species: importance and applications for plant population genetics, evolution, and biotechnology. Front Plant Sci.

[B19] Santos da Silva R, Clement CR, Balsanelli E, Baura VA, Souza EM, Fraga HPF, do Nascimento Vieira L (2021). The plastome sequence of Bactris gasipaes and evolutionary analysis in tribe Cocoseae (Arecaceae). PLoS One.

[B20] Velazco SJE, Svenning JC, Ribeiro BR, Laureto LMO (2020). On opportunities and threats to conserve the phylogenetic diversity of Neotropical palms. Divers Distrib.

[B21] Zhu A, Guo W, Gupta S, Fan W, Mower JP (2016). Evolutionary dynamics of the plastid inverted repeat: The effects of expansion, contraction, and loss on substitution rates. New Phytol.

