# Supplementary material for: The complete plastid genome of Bactris riparia (Arecaceae) and a comparative analysis in Bactridinae (Cocoseae, Arecaceae)
Source: Genet Mol Biol. 2022 Nov 7;45(4):e20210305. doi: 10.1590/1678-4685-GMB-2021-0305 (PMC9641374; doi:10.1590/1678-4685-GMB-2021-0305)
Supplement: Table S1 - [file 1415-4757-GMB-45-4-e20210305-s1.pdf]

Genetics and Molecular Biology
https://doi.org/10.1590/1678-4685-GMB-2021-0305

Supplementary Material to "The complete plastid genome of *Bactris riparia* (Arecaceae) and a comparative analysis in Bactridinae (Cocoseae, Arecaceae)"

Table S1 - Non-synonymous (Ka), synonymous (Ks), and Ka/Ks ratio of Bactridinae protein-coding genes.

Genes	Ka	Ks	Ka/Ks
accD	0	0,01	0,54
atpA	N/A	0,01	N/A
atpB	N/A	0,01	N/A
atpF	0	0	0,29
atpH	N/A	0,01	N/A
atpI	0	0	0,22
ccsA	0,01	0	1,82
cemA	0	0	1,95
clpP	0	0	0,3
infA	N/A	0,01	N/A
matK	0	0	0,66
ndhA	0	0	0,4
ndhB	N/A	0	N/A
ndhD	0	0	0,45
ndhF	0	0,01	0,6
ndhG	N/A	0	N/A
ndhH	N/A	0	N/A
ndhI	N/A	0	N/A
ndhJ	0	0,01	0,12
ndhK	0	N/A	N/A
petA	0	N/A	N/A
petB	N/A	0	N/A
petD	N/A	0	N/A
petN	N/A	0,02	N/A
psaA	N/A	0,01	N/A
psaB	0	0	0,05
psaI	N/A	0,02	N/A
psaJ	N/A	0,02	N/A
psbA	0	0,01	0,04
psbB	0	0,01	0,12
psbC	N/A	0,01	N/A
psbK	N/A	0,01	N/A
psbT	N/A	0,01	N/A
rbcL	0,01	0,01	0,73
rpl16	0,01	N/A	N/A
rpl20	N/A	0,01	N/A

**Genes**	**Ka**	**Ks**	**Ka/Ks**
rpl22	0	0,01	0,29
rpl32	N/A	0,04	N/A
rpoA	N/A	0	N/A
rpoB	0	0	0,24
rpoC1	0	0	1,03
rpoC2	0	0,01	0,26
rps2	0	0,01	0,45
rps3	0	0,01	0,32
rps4	N/A	0,01	N/A
rps8	0	N/A	N/A
rps11	0	0	0,37
rps15	0	0,02	0,21
rps18	0	N/A	N/A
ycf1	0,01	0,01	0,77
ycf3	0	N/A	N/A
ycf4	0	0	0,21
